# Segmental Additive Tissue Engineering

**DOI:** 10.1038/s41598-018-29270-4

**Published:** 2018-07-18

**Authors:** Martina Sladkova, Rawan Alawadhi, Rawan Jaragh Alhaddad, Asmaa Esmael, Shoug Alansari, Munerah Saad, Jenan Mulla Yousef, Lulwa Alqaoud, Giuseppe Maria de Peppo

**Affiliations:** The New York Stem Cell Foundation Research Institute, New York, NY USA

## Abstract

Segmental bone defects caused by trauma and disease represent a major clinical problem worldwide. Current treatment options are limited and often associated with poor outcomes and severe complications. Bone engineering is a promising alternative solution, but a number of technical challenges must be addressed to allow for effective and reproducible construction of segmental grafts that meet the size and geometrical requirements needed for individual patients and routine clinical applications. It is important to devise engineering strategies and standard operating procedures that make it possible to scale up the size of bone-engineered grafts, minimize process and product variability, and facilitate technology transfer and implementation. To address these issues, we have combined traditional and modular tissue engineering approaches in a strategy referred to as Segmental Additive Tissue Engineering (SATE). To demonstrate this approach, a digital reconstruction of a rabbit femoral defect was partitioned transversally to the longitudinal axis into segments (modules) with discoidal geometry and defined thickness to enable protocol standardization and effective tissue formation *in vitro*. Bone grafts corresponding to each segment were then engineered using biomimetic scaffolds seeded with human induced pluripotent stem cell-derived mesodermal progenitors (iPSC-MPs) and a novel perfusion bioreactor with universal design. The SATE strategy enables the effective and reproducible engineering of segmental bone grafts for personalized skeletal reconstruction, and will facilitate technology transfer and implementation of a tissue engineering approach to segmental bone defect therapy.

## Introduction

Repair of segmental skeletal defects resulting from trauma and disease still remains an elusive procedure that requires long periods of time, high costs, and significant efforts by patients and surgeons. Treatment options are limited, particularly for pediatric patients, and these are often associated with poor outcomes and severe complications^1^. Novel tissue engineering approaches create the possibility that viable bone grafts could be grown on demand^2^, and thus represent a promising alternative solution for personalized reconstruction of segmental skeletal defects^3^. Over the last several years, researchers have successfully grown bone grafts^4^ with customized shapes^5^, which have shown promising outcomes in animal models of skeletal defects^6–9^. However, there remain manufacturing-related challenges to the effective and reproducible construction of viable segmental bone grafts with the specific size and geometrical requirements needed for individual patients and routine clinical applications. These challenges include the need for large amounts of bone-forming cells, uniform seeding of large and anatomically-shaped scaffolds, long distance perfusion of segmental grafts in bioreactors, customization of bioreactor design and, most importantly, process standardization. These factors limit the size of bone grafts that can be engineered in the laboratory and hamper implementation of the technology for use in the clinic. It is therefore critical to devise novel engineering strategies that minimize process and product variability, and facilitate transition of tissue-engineered segmental bone grafts from bench to bedside. To overcome these manufacturing challenges, the current study combines traditional and modular tissue engineering approaches in a strategy referred to as Segmental Additive Tissue Engineering (SATE), and state-of-the-art cell and tissue culture technologies towards generating segmental bone grafts effectively and reproducibly. To demonstrate this approach, we have partitioned a digital reconstruction of a 3-cm long rabbit femoral defect transversally to the longitudinal axis into segments (modules) with discoidal geometry and defined thickness. The discoidal geometry of each segment enables the standardization of seeding procedures and perfusion conditions in the bioreactor, while its restricted thickness facilitates effective tissue formation *in vitro*. We have then engineered bone grafts corresponding to each segment using biomimetic decellularized cow bone scaffolds seeded with human induced pluripotent stem cell-derived mesodermal progenitors (iPSC-MPs) and a novel perfusion bioreactor with universal design.

## Results and Discussion

**Figure 1:**
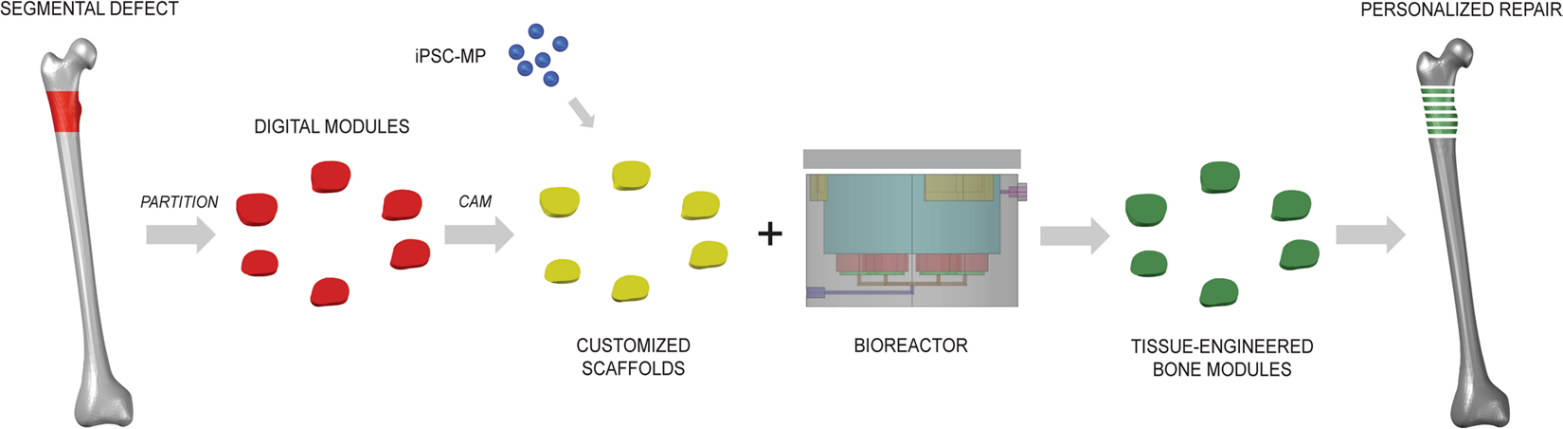
Segmental additive tissue engineering (SATE). Digital reconstructions of segmental bone defects (red region) are partitioned transversally to the longitudinal axis, resulting in the formation of segments (modules) with discoidal geometry and defined thickness. Following partition, the digital segments are used to assist the manufacturing of customized scaffolds and perfusion inserts for tissue culture in a novel SATE perfusion bioreactor with universal design. The tissue-engineered segments (bone modules) are finally pieced together for personalized reconstruction of segmental bone defects. Abbreviations = iPSC-MP, induced pluripotent stem cell-derived mesenchymal progenitors.

Bone engineering is anticipated to provide better treatments for patients suffering from segmental bone loss caused by trauma and disease. However, despite the numerous attempts reported, there remain significant engineering and manufacturing challenges in order to transition from a research scale to a clinically applicable production of therapeutically safe and effective tissue-engineered bone grafts. Growing segmental bone grafts in the laboratory presents several issues that can affect product consistency and hinder technology transfer and implementation. By partitioning digital reconstructions of segmental defects into modules, some of these issues may be overcome, facilitating the transition of bone-engineered segmental grafts to the clinic. Here we report the benefits and advantages of the SATE strategy (outlined in Fig. 1 and Video S1), and provide standard operating procedures that will lead towards the reproducible and effective engineering of segmental bone grafts.

### Partitioning of defect reconstructions and assisted manufacturing of perfusion inserts and scaffolds

**Figure 2:**
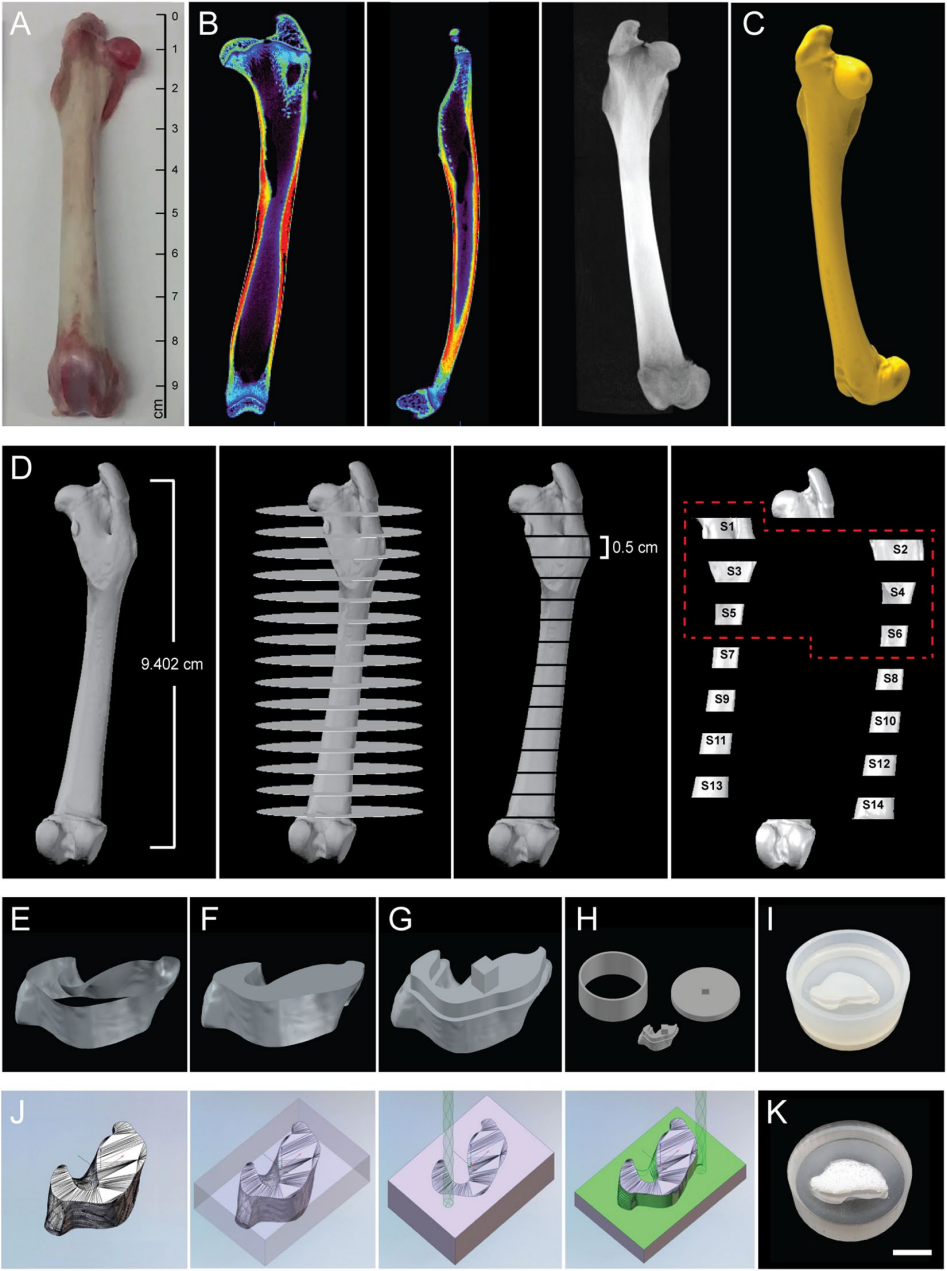
Partitioning of bone defect reconstructions and manufacturing of perfusion inserts and scaffolds for culture in bioreactor. (**A**) Photograph of a rabbit femur (right leg). (**B**) Image data of the femur (virtual femur) visualized in ASIPro VM™ (front view, side view and 3D) (**C**) 3D model of the virtual femur as reconstructed in Simpleware. (**D**) Partition of the rabbit femur into 14 virtual segments (S1-S14) with discoidal geometry and defined thickness (0.5 cm each). Only the first six virtual segments were used for SATE (red dashed line) (**E**) View of virtual segment 1 generated from partitioning of the digital model of the rabbit femur. (**F**) Patched segment 1. (**G**) Segment 1 reshaped in AutoCAD to include an offset and a plug for the construction of an equilibration chamber within the perfusion insert. (**H**) Digital representation of a three-part mold for casting the perfusion insert corresponding to segment 1. (**I**) Assembled 3D printed mold filled with PDMS for the construction of the perfusion insert corresponding to segment 1. (**J**) Simulation of the milling process for the construction of a customized scaffold corresponding to virtual segment 1. (**K**) 5-axis milled decellularized bone scaffold corresponding to segment 1 placed inside its respective perfusion insert. Abbreviations: 3D, three-dimensional; S, segment; PDMS, polydimethylsiloxane. Additional data are shown in Video S2, Figs S1, S2, S3, S4, S5 and S6.

In anticipation of future testing in animal models of skeletal defects, we have engineered a segmental bone graft corresponding to a defect occurring in the proximal region of a rabbit femur (representing about 30% of the total femur volume). To do this, we scanned the femur of an adult rabbit using micro-computed tomography (µCT) (Fig. 2A) and generated DICOM files (Fig. 2B and Video S2) to build a high fidelity surface model (Fig. 2C) of the bone. We then partitioned the model into independent segments (Fig. 2D,E) and used them as templates to assist the manufacturing of perfusion inserts and customized scaffolds for culture in a novel SATE perfusion bioreactor with universal design. A segment thickness of 0.5 cm was chosen in this study based on previous experience in our laboratory^10^. However, the maximum segment thickness that the SATE strategy allows to be engineered depends on the scaffold architecture and perfusion regime adopted. Thus, the thickness of each segment could be increased to larger values under optimized culture conditions. In this study we used the proximal six segments generated from partitioning of the bone digital reconstruction, corresponding to a 3-cm long rabbit femoral defect. Following partitioning, we patched the surface models of individual segments to produce 3D objects (Figs 2F and S1) and used these as templates to manufacture customized perfusion inserts and scaffolds. Based on the simulation studies (Fig. S3), and to facilitate development of a SATE perfusion bioreactor with universal design (described in the next paragraph), we have modified the geometry of each segment (Fig. 2G) to create a flat-bottom equilibration chamber (with a 2 mm reduction in diameter) within the perfusion inserts holding the scaffolds (Figs 2H,I, S4, S5 and S6). The equilibration chamber allows redistribution of the medium exiting the channels, thereby facilitating uniform perfusion of the grafts irrespective of their size and geometry (Fig. S3).

Similarly, we have used the digital models of each segment as templates to manufacture customized decellularized cow bone scaffolds via computer numerical control (CNC) milling (Fig. 2J), in order to position the scaffolds in a press-fitted manner into their corresponding perfusion inserts (Fig. 2K). This configuration enables direct tissue perfusion during culture in the SATE bioreactor, a dynamic condition that is recognized to be the most suitable to support effective growth of tissue-engineered bone *in vitro*^11^. Alternatively, the digital files could be used to assist the fabrication of different biomaterial scaffolds via other manufacturing techniques such as rapid prototyping and casting technologies. The use of synthetic materials, such as for example ceramic- and polymer-based scaffolds and combination thereof, is expected to foster translation of tissue-engineered products, and could be employed for growing segmental bone grafts using the SATE strategy. The ability to tune the architectural characteristics of biomaterials scaffolds will facilitate optimization of seeding protocols and perfusion conditions, and will thus increase reproducibility in segmental bone engineering applications using the SATE strategy.

### SATE perfusion bioreactor

**Figure 3:**
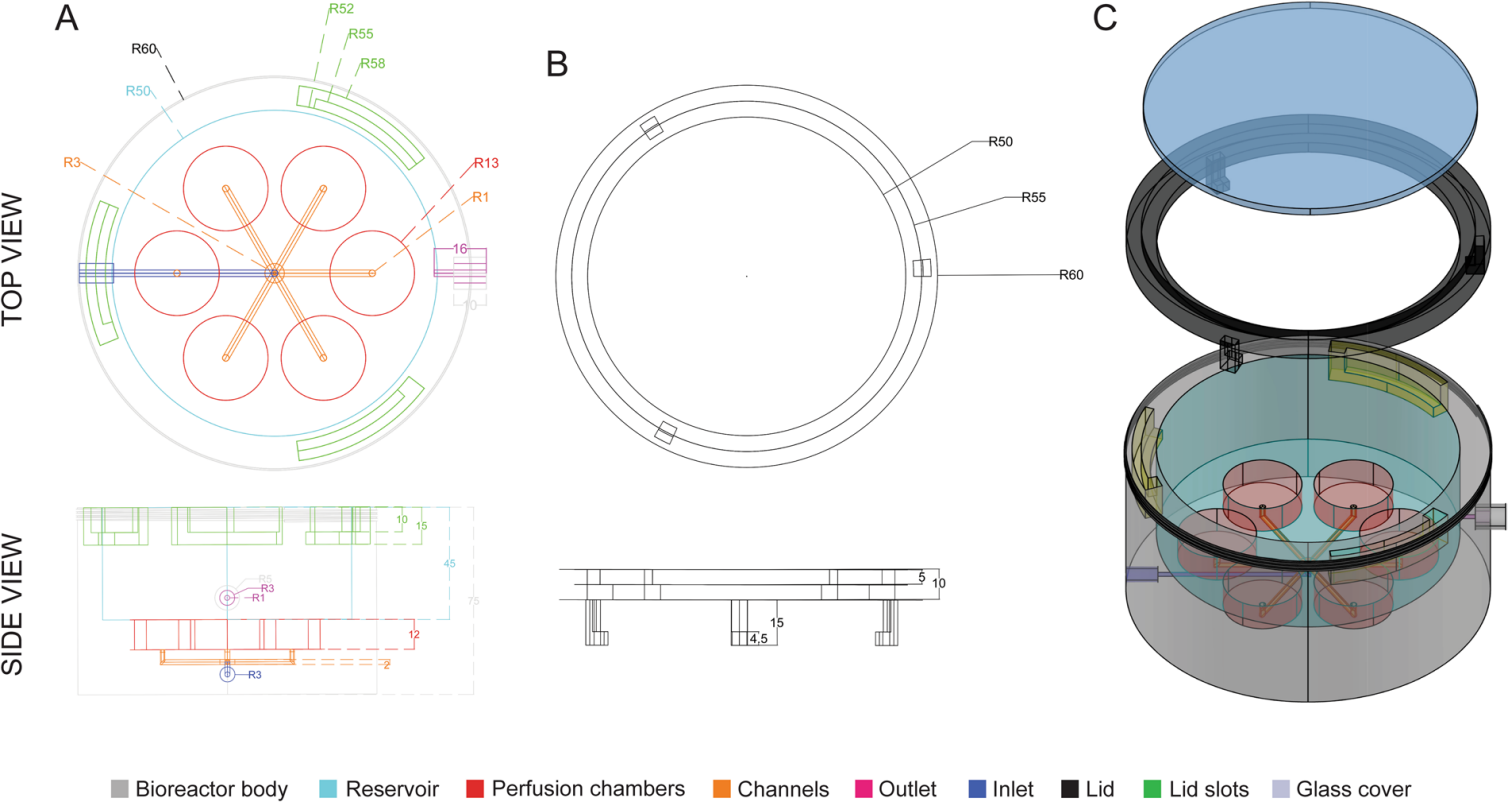
SATE perfusion bioreactor. (**A**) Wireframe top and side view of the SATE bioreactor body. (**A**) Wireframe top and side view of the SATE bioreactor lid frame. (**C**) Isometric x-ray view of the SATE bioreactor, lid frame and cover glass. Different colors represent different SATE bioreactor elements. Dimensions are in mm. Abbreviations: R, radius. Additional data are shown in Videos S3 and S4, Table S1, Figs S7 and S8.

Over the last two decades, bioreactors have created new possibilities in the field of bone engineering as they support tissue formation *in vitro* by providing an appropriate physiological environment^11^. Most importantly, bioreactors support the efficient delivery of nutrients to cells during culture and should therefore make it possible to engineer large volume bone grafts. However, despite the multitude of studies reported, bioreactors for bone engineering remain at a prototype phase, with practically no bioreactors available on the market^11^. Customization of bioreactor design to meet specific clinical needs is in fact technically challenging, expensive, and time consuming, thus limiting technology transfer and implementation, as well as delivery of bone-engineered products to patients. To address these challenges, we have developed a perfusion bioreactor with a universal design (Fig. 3 and S6, Video S3 and Table S1), i.e. with a configuration suitable for generating segmental bone grafts with a broad range of sizes and geometries. The bioreactor is a self-contained, simple design consisting of an inlet, a system of channels (manifold), six tissue chambers (corresponding to the number of segments engineered in this study), a reservoir, a body, an outlet and a lid for easy access and monitoring of the culture environment (Fig. 3 and S7, Videos S3 and S4). The tissue chamber diameter is equivalent to the diameter of the perfusion inserts holding the grafts in a press-fitted manner to allow direct tissue perfusion during culture (Fig. S8). The number of tissue chambers as well as their volume can be changed as needed to meet specific experimental or clinical needs. To account for large differences in graft diameter, each tissue chamber could be isolated and perfused independently. This way, the perfusion load can be regulated so that each segment is subjected to comparable shear stress during culture to minimize product variability. In order to reduce manufacturing time and allow production at affordable cost, we have come up with a design suitable for manufacturing via 3D printing. To meet the requirements for clinical translation, we have chosen a biocompatible material (Nylon) that does not release toxic products, displays good chemical resistance and low moisture absorption, and withstands autoclaving.

### Cell seeding on scaffolds and culture in the SATE bioreactor

Growing functional tissues in the laboratory requires an ample supply of cells as building blocks supporting histogenesis. It is estimated that hundreds of millions of cells are needed to grow bone grafts for segmental skeletal reconstructions. Traditional attempts to grow cm-sized bone grafts have used mesenchymal stem cells derived from adult tissues as source of bone forming cells. However, these cells proliferate slowly, rapidly reach senescence, and may not be available in sufficient number for every patient^3,12^. In contrast, recent developments in nuclear reprogramming allow the generation of virtually unlimited numbers of autologous cells that have the ability to differentiate into all of the specialized cells constituting the bone tissue^13^. This creates the possibility that segmental bone grafts may be grown on demand for personalized clinical applications. Recently, we have derived mesodermal progenitor cells from human iPSCs and engineered functional bone tissue using a biomimetic approach to bone development^10^. In this study, we have demonstrated that a similar approach can be applied toward the effective and reproducible construction of segmental bone grafts. Previous studies in our laboratory have shown that iPSC-MPs can be expanded to trillion of cells in just a few weeks time period^10,14^, thus allowing the engineering of segmental bone grafts for autologous reconstruction at a reasonable cost and in a relatively short period of time.

**Figure 4:**
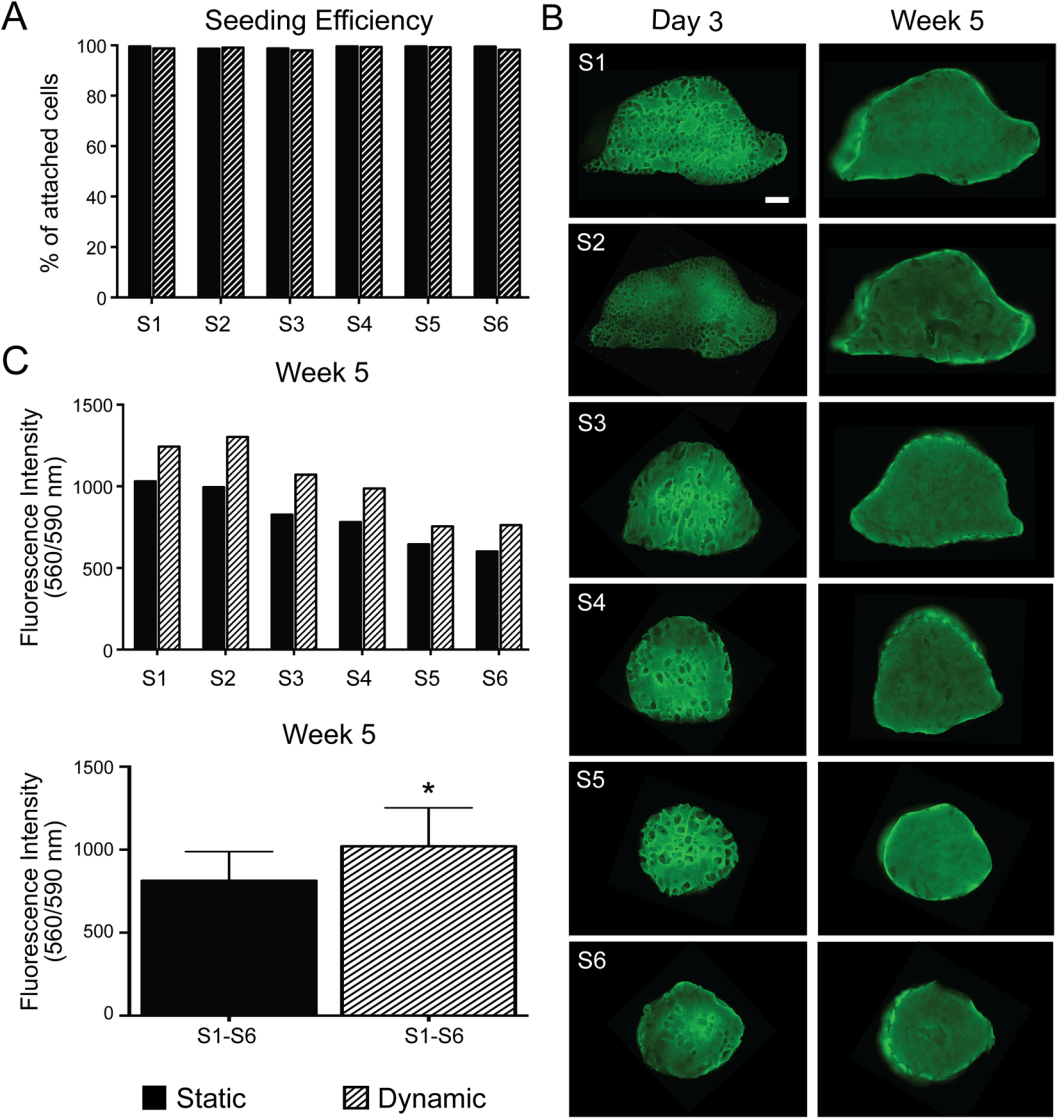
Cell seeding and growth on scaffolds. (**A**) Percentage of attached 1013A-derived mesenchymal progenitors (1013A-MPs) 1 day after seeding onto decellularized bone scaffolds corresponding to the six defect segments (S1–S6). (**B**) Epifluorescence mosaic micrographs showing distribution and viability of 1013A-MPs stained with fluorescein diacetate (FDA; green) onto decellularized bone scaffolds corresponding to the six defect segments (S1–S6) 3 days after seeding, and 5 weeks after culture under dynamic conditions in the SATE bioreactor. Scale bar: 2 mm. (**C**) Quantification of 1013A-MPs seeded onto decellularized bone scaffolds corresponding to the six defect segments (S1-S6) 5 weeks after culture under static or dynamic conditions in bioreactors. Data represent averages ± SD (n = 6), Student’s t-test, P < 0.05; *denotes significant difference to static conditions. Abbreviations: S, segment. Additional data are shown in Table S2 and Figure S9.

A critical step in tissue engineering involves the seeding of cells on biomaterial scaffolds. In fact, it has been shown that the number of cells as well as their spatial distribution in the scaffolds can influence migration, communication and differentiation^15^, and affect the quality and regenerative properties of tissue-engineered products. The efficiency of cell seeding is affected by many variables and for the most part by scaffold architecture, size and geometry^16^. Therefore, optimization and standardization of cell seeding on scaffolds is a crucial requirement for the reproducible *in vitro* cultivation of functional bone grafts, especially if tissue-engineered products are to be used in the clinic to treat patients. Partitioning of segmental defect reconstructions into segments with discoidal geometry enables standardization of the seeding protocol, thereby resulting in the effective and reproducible growth of bone grafts for segmental reconstruction. In this study, we have used decellularized cow bone as scaffolding material because of its good mechanical properties, which are important for segmental reconstructions in load bearing locations, and because of existing knowledge on its potential to support the construction of bone grafts from different cell sources^5,9,10^. The scaffolds were seeded with the cells while they were placed within their respective perfusion inserts (Fig. S8A) in order to increase seeding efficiency and reduce variability between samples. Cell seeding on scaffolds using our optimized protocol led to nearly 100% attachment with highly uniform distribution of cells irrespective of the size and geometry of the scaffolds (Figs 4A,B and S9). The number of cells (Table S2) and the volume of cell suspension used per scaffold were kept consistent. After 5 weeks under osteogenic conditions, samples cultured in the SATE perfusion bioreactor display an increased number of viable cells as compared to samples cultured in static conditions (Fig. 4C), despite the similar seeding efficiency observed (Fig. 4A), highlighting the importance of a dynamic system for effective engineering of large bone grafts.

### Tissue formation and graft assembly

**Figure 5:**
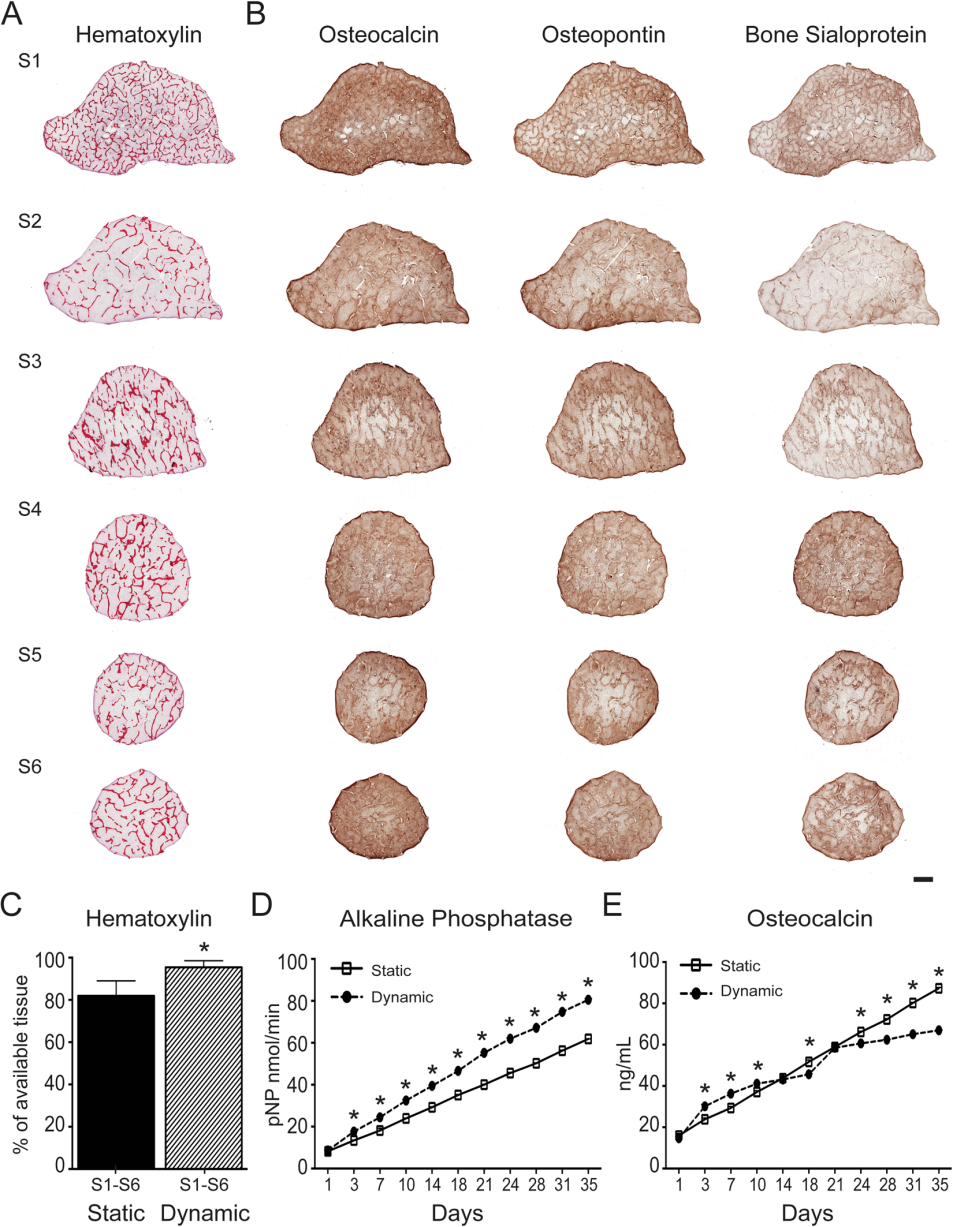
Tissue formation. (**A**) Histological analysis of samples corresponding to the six defect segments (S1-S6) cultured under dynamic conditions in bioreactors. Samples are stained with hematoxylin/eosin, which stains cell nuclei blue and extracellular matrix pink. Scale bar: 2 mm. (**B**) Immunohistochemical analysis of samples corresponding to the six defect segments (S1-S6) cultured under dynamic conditions in bioreactors. Samples stain positive (brown) for osteocalcin, osteopontin and bone sialoprotein. Scale: 2 mm. (**C**) Quantification of hematoxylin/eosin staining for samples corresponding to the six defect segments (S1-S6) cultured under static and dynamic conditions in bioreactors. Data represent averages ± SD (n = 18, Student’s t-test, P < 0.05; *denotes significant difference to static conditions). (**D**) Quantification of alkaline phosphatase and osteocalcin released from samples cultured under static and dynamic conditions in bioreactors. Data represent averages ± SD (n = 3, Student’s t-test, P < 0.05; *denotes significant difference to static conditions). Abbreviations: S, segment. Additional results are presented in Figs S10 and S11.

Effective growth of bone grafts in the laboratory must lead to the formation of viable tissues with a compact extracellular matrix characterized by the presence of bone-specific proteins. We have previously reported that perfusion culture is critical for engineering compact bone grafts from human iPSC-derived mesodermal progenitors^10^. Therefore, in this study we applied the same osteoinductive and perfusion conditions as previously described. Histological and immunohistochemical analysis of samples reveal excellent tissue formation during culture as evidenced by progressive cell penetration and synthesis of bone-specific extracellular matrix (Fig. 5A,B). Importantly, the analyses reveal higher tissue formation (Figs 5A–C, S10A,B and S11) and release of alkaline phosphatase (Fig. 5D) when the cell-scaffolds constructs are cultured in the SATE perfusion bioreactor as compared to static controls, supporting the vital role of interstitial flow for cell survival and differentiation, and tissue formation. Under dynamic conditions there is uniform tissue formation throughout the whole constructs irrespective of their diameter and shape. These results reflect the data obtained from the *in silico* studies, and highlight the importance of protocol standardization for the effective and reproducible production of tissue-engineered grafts.

Modular approaches of tissue engineering had previously been reported for scale up of the size of tissue-engineered bone grafts^4,6^. However, assembly of small tissue modules results in the formation of segmental grafts with poor integrity and limited structural capability. On the other hand, partitioning of segmental defect reconstructions using the SATE strategy results in the formation of tissue modules that can be assembled into segmental grafts with high mechanical stability. Biocompatible bone adhesives or orthopedic devices such as external fixators, intramedullary nails, and osteosynthesis plates could ultimately be used to provide sufficient stability and maintain the alignment of the tissue modules engineered using the SATE strategy during reconstruction and healing.

Importantly, the SATE strategy is also expected to facilitate development of *in vitro* pre-vascularization protocols, enable cryopreservation, and expedite neovascularization following implantation.

## Conclusions

Attempts to engineer segmental bone grafts have been reported over the past years using different tissue engineering strategies. However, these strategies do not address the need for process standardization, which is critical to enable technology transfer and implementation toward the effective and reproducible construction of segmental bone grafts. In this study, we have addressed several manufacturing-related issues and devised standard operating procedures that could foster clinical translation of tissue-engineered segmental bone grafts. The SATE strategy is highly versatile and easily implemented, and shows a real potential to improve the quality of life of pediatric and adult patients suffering from segmental bone defects.

## Materials and Methods

### Partitioning of segmental bone defects

Rabbit legs were purchased (D’Artagnan Inc., Newark, NJ) and cleaned to remove the skin and soft tissue and expose the bone. Image data of the right femur were obtained using a microcomputed tomography (µCT) scanner (Siemens Medical Solutions USA Inc., Malvern, PA) and visualized in ASIPro VM™ (Siemens Medical Solutions). DICOM (Digital Imaging and Communications in Medicine) files were then imported to Simpleware ScanIP (Simpleware, Exeter, UK) for image processing and three-dimensional (3D) model generation. Briefly, a mask was created for visualization using a threshold with range values from 837 to 5422. Following smoothing of the mask, a model of the femur was generated by meshing the full model using the finite element module. Finally, the NURBS (non-uniform rational basis spline surface) was exported as IGES (initial graphics exchange specification) file and converted into a drawing file format using Autodesk Inventor (Autodesk, San Rafael, CA). The digital model of the rabbit femur was finally loaded in a computer-aided design (CAD) program (AutoCAD, Autodesk) for partitioning. Briefly, 0.2 mm thick cylinders were subtracted from the model resulting in the generation of 14 segments, each with a thickness of 0.5 cm. The segments were then isolated and patched to create 3D objects for computer-aided manufacturing (CAM) of customized perfusion inserts and decellularized bone scaffolds. The proximal six segments generated from partitioning were used in this study (S1 to S6), corresponding to a 3 cm long femoral defect.

### *In silico* simulation

DICOM files generated from µCT of decellularized bovine bone scaffolds (4 mm height and 4 mm in diameter), were imported in ScanIP Simpleware (Simpleware) for generation of a 3D model and subsequent creation of a cross section image along the longitudinal axis. Following the conversion of the 2D image from JPEG to DWG file format using Img2CAD 7.2 (Img2CAD, Cologne, Germany), the file was imported in AutoCAD (Autodesk) to draw a close replica of the 2D image and reproduce a larger image, with a size corresponding to the diameter of the larger segment (S1) generated from partitioning of the rabbit femoral model. Thus, the digital drawing was placed into a perfusion system framework characterized by an inlet (8 mm length and 3 mm width), an equilibration chamber (with flat or tapered geometry of variable size), a perfusion chamber (4 mm length and 20 mm width) and an outlet (10 mm length and variable width: 15.55–17.55). The hydrodynamic environment was then simulated using COMSOL Multiphysics (COMSOL, Inc., Palo Alto, CA) to get recommendations on the effect of bioreactor design and inlet velocity on fluid dynamics. The geometry was imported in COMSOL Multiphysics as a DXF (drawing exchange format) file with the scaffold area set to bone material. Wall parameters were set with a boundary condition of no slip. Given the complexity of the scaffold architecture and to reduce computational time the model was meshed using normal tetrahedral elements. Due to the low Reynolds’s number (<2000) at selected inlet fluid velocities (5.75E-9–5.75E-5 m/s), a Navier-Stokes physics for steady-state laminar flow was used for simulation (1):

1 $$\rho (v\cdot \nabla )v=\nabla \cdot [-pl+\mu (\nabla v+{(\nabla v)}^{{\rm T}})]+{\rm{F}}$$

where *v* is the fluid velocity, *p* is the fluid pressure, *ρ* is the fluid density, and *μ* is the fluid dynamic viscosity^17^.

### Design and manufacturing of molds for casting the perfusion inserts

The molds for the production of customized perfusion inserts were designed in AutoCAD (Autodesk). Different designs were tested to enable production of flawless perfusion inserts. The most optimal configuration consisted of a 3-part design characterized by a base (29 mm in diameter), a ring (29 mm outer diameter, 26 mm inner diameter and 10 mm height) and the segment joined together. To comply with the simulation results and build an equilibration chamber within the perfusion inserts, the surface edge of each segment model (S1-S6) was retraced in AutoCAD (Autodesk) through the use of spline. The spline was offset 1.5 mm from the edge of each segment, and extruded 2 mm to create the negative for the production of the equilibration chamber within the perfusion inserts. On top of the extruded object a cubic plug (1.8 × 1.8 mm) was added to secure the retraced segment to the base of the mold (via an opening of 2 × 2 mm drawn in its center). The mold parts were finally exported as STL (stereolithography) file and 3D printed by Proto Labs (Proto Labs, Inc., Maple Plain, MN) using the Somos® 9120 photopolymer.

### Manufacturing of customized perfusion inserts

The customized perfusion inserts were manufactured via casting using polydimethylsiloxane (PDMS). Briefly, SYLGARD 184 elastomer (Dow Corning, Midland, MI) base was mixed with the curing agent (Dow Corning) at a ratio of 9:1 (w/w) in a 50 ml conical centrifuge tube. After thoroughly mixing the elastomer base and curing agent using a metal spatula, the mixture was placed under vacuum to degas. The mixture was finally poured into the assembled molds, and cured for 1 hour at 60 °C. After cooling overnight, the perfusion inserts were carefully removed from the molds and cleaned.

### Manufacturing of customized biomaterial scaffolds

Decellularized bone scaffolds were prepared as previously described^10^. Briefly, blocks of trabecular bone were sawed from the proximal and distal regions of cow legs. Soon after, the blocks were power washed to remove the bone marrow, then sequentially treated with a solution of 0.1% (w/v) ethylenediaminetetraacetic acid (EDTA; Sigma #E6758-100G) in phosphate buffer saline solution (DPBS; w/v), 0.1% (w/v) EDTA in 10 mM Tris-HCL buffer (10 mM; Fisher Scientific #BP1757-500), and 0.5% (w/v) sodium dodecyl sulfate (SDS; Sigma #L3771-100G) in 10 mM Tris-HCL buffer, followed by treatment with a solution of DNase (100 U/mL; Sigma-Aldrich #D4263-5VL) and RNase (Sigma-Aldrich #R4875-100MG) in distilled H_2_O to remove cellular and genetic material. Decellularized bone blocks were thoroughly rinsed in DPBS, freeze-dried, and processed into the final shape using a 5-axis milling machine at B&J Specialty (B&J Specialty, Inc, Wawaka, IL). Before seeding, each customized scaffold was placed in the respective PDMS chamber, sterilized overnight in 70% (v/v) ethanol, and conditioned in expansion medium for 24 hours.

### Bioreactor design, manufacturing and validation

The SATE perfusion bioreactor with universal design was drawn in AutoCAD (Autodesk). To ease technology transfer and enable manufacturing via 3D printing, the bioreactor was designed by drawing the perfusion system first, i.e. ports, channels or manifold, perfusion chambers and reservoir, and then subtracting the whole perfusion system from a single overlapping 3D object. Initially, a cylinder was drawn to create the reservoir. Underneath the reservoir 6 cylinders distributed on an array were drawn to create the perfusion chambers. Beneath each perfusion chamber a L-shaped channel was drawn to integrating all perfusion chambers into a manifold hub. Finally, from the manifold hub center, another L-shaped channel was drawn terminating as an inlet port. Following subtraction of the perfusion system from an overlapping 3D object (in this case a cylinder), a hole was drawn on the wall of the reservoir to produce the outlet port. Both the inlet and outlet ports were threaded for securing the connectors to the tubing system. After drawing the main body, a lid was designed to maintain sterility while enabling noninvasive monitoring of the culture environment during tissue growth. In one proposed configuration, the lid was designed as a cylinder frame with a clear glass ceiling. In order to secure the lid to the bioreactor main body, a hook-slot mechanism was adopted. Briefly, three slots were created inside the wall of the bioreactor body and three hooks at corresponding locations were added to the lid frame. The bioreactor and lid designs were saved as STL files and 3D printed at Stratasys (Stratasys, Ltd, Eden Prairie, MN) in Nylon 12 PA. The borosilicate glass (McMASTER-CARR, #8477K37; 4-1/4” in diameter, 1/8” in length) was attached to the lid frame using an adhesive for extreme conditions (Loctite, Düsseldorf, Germany). The bioreactor was then connected to the tubing system using leakage proof flangeless connectors (Tefzel™ ETFE, IDEX Health & Science, Middleborough, MA). Uniform fluid perfusion across the channels system/manifold, and simultaneously through each perfusion chamber, was confirmed before the experiment.

### Construct assembly and culture in perfusion bioreactor

Induced pluripotent stem cell-derived mesenchymal progenitors (line 1013 A) were derived and characterized as previously described^10^. Cells were expanded in medium consisting of high-glucose KnockOut Dulbecco’s Modified Eagle’s Medium (KO-DMEM; Gibco, Grand Island, NY), 10% (v/v) HyClone fetal bovine serum (GE Life Sciences, Pittsburgh, PA), beta-fibroblast growth factor (1 ng/ml; R&D systems, Minneapolis, MN), GlutaMax (1X; Gibco), non essential amino acids (1X; Gibco), 0.1 mM β-mercaptoethanol (Gibco), and antibiotic-antimycotic (1X; Gibco). At passage 5, cells were detached using trypsin (0.25% Trypsin EDTA; Fisher Scientific, #25200-056) and counted using a hemocytometer. Before seeding, each scaffold was placed in the respective perfusion insert and sterilized in 70% (v/v) ethanol overnight. Then after, the scaffolds were conditioned in expansion medium for 24 hours, blot-dried using autoclaved Kimwipes (Fisher Scientific), transferred to ultra-low attachment plates (Fisher scientific, #7200601) and seeded with cells using a droplet technique. Following seeding, constructs were placed in a humidified environment at 37 °C for 3 hours to let cells attach on the scaffolds, and then transferred to an ultra-low attachment 6-well plate containing expansion media. The following day, the expansion medium was collected and non-adherent cells counted using a hemocytometer to estimate the seeding efficiency. Following culture in expansion medium for 3 days, the cell-scaffold constructs were transferred to the perfusion bioreactor and cultured in osteogenic medium consisting of high-glucose DMEM (Thermo Fisher Scientific, #11965-092) supplemented with 10% (v/v) HyClone fetal bovine serum (Thermo Fisher Scientific, #SH30071.03), L-ascorbic acid (50 µM; Sigma-Aldrich, #A8960-5G), dexamethasone (1 µM; Sigma-Aldrich), and β-glycerophosphate disodium salt (10 mM; Sigma-Aldrich) for 5 weeks. The samples were perfused with a uniform flow rate of 3.6 ml/min using a digital, low-flow, multichannel Masterflex peristaltic pump (Cole Palmer). Control samples were removed from the perfusion frameworks and cultured in static conditions in 6-well ultra-low attachment plates (Fisher Scientific). At the end of the culture, the samples were harvested and processed for biochemical and histological analyses.

### Cell distribution, viability and growth on scaffolds

Cell distribution, viability and growth on scaffolds were determined using qualitative and quantitative methods. To investigate cell distribution, 3 days after seeding and 5 weeks after culture in osteogenic induction conditions, the samples were stained using fluorescein diacetate (Thermo Fisher Scientific). Working reagent of fluorescein diacetate (FDA) was prepared as follows: 20 mg of FDA powder was diluted in 100% acetone, then diluted in DPBS at a ratio of 1:1, and filtered. Staining was performed with 20 µL of working reagent per 1 ml of expansion medium, incubated 10 min at 37 °C in the dark, and imaged in RPMI medium (Life Technologies, #118365-030) using the Olympus IX71 epifluorescence microscope (Olympus) with blue excitation filter. In order to obtain images of the whole samples, mosaic pictures were assembled from sequential fluorescent images using the ImageJ (NIH) software equipped with the MosaicJ and TurboReg plugins.

The amount of cells present in the samples at the end of the culture period was estimated using the PrestoBlue™ assay. Briefly, samples were treated with 6 ml of osteogenic medium containing 10% (by volume) of PrestoBlue™ reagent (Life Technologies) and incubated for 2 hours at 37 °C. Then after, aliquots of culture media (200 µl) were transferred to a black, clear, flat-bottom 96-well plate (BD Falcon^TM^) and fluorescence measured at 560/590 nm (excitation/emission) using the fluorescent reader SYNERGYMx (BioTek) equipped with Gen 5 1.09 software. The amount of viable cells was expressed as intensity of measured fluorescence.

### ALP activity

The release of alkaline phosphatase (ALP) into culture media was monitored every three days during the entire culture period using the Alkaline Phosphatase Colorimetric Assay Kit (BioVision, Milnitas, CA, #K412-500) according to the manufacturer’s instructions. At each media change, samples were collected from both the SATE bioreactor and the static cultures and used for the analysis. Briefly, 80 µl aliquots of culture media were added to a transparent, flat-bottom, 96-well plate, mixed with 50 µl of 5 mmol paranitrophenylphosphate (*p*NPP) diluted in ALP Assay Buffer, and incubated at room temperature for 2 hours. The reaction was stopped with 20 µl of Stop Solution and absorbance was read at 405 nm using the plate reader SYNERGYMx (BioTek) equipped with Gen 5 1.09 software. The ALP activity (i.e. the amount of produced paranitrophenol [*p*NP] per sample) was calculated by subtracting background readings and using the *p*NPP standard curve. The results were expressed as “ALP cumulative release” in µmol/h.

### Osteocalcin release

The amount of osteocalcin released into culture media was analyzed every 3 days during the entire culture period (5 weeks) using the Gla-type Osteocalcin EIA kit (Takara, Mountain View, CA, #MK111) according to the manufacturer’s protocol. At each media change, medium samples were collected as described in the **Alkaline phosphatase activity** section for analysis. Briefly, the Gla-type Osteocalcin EIA plate was incubated with 100 µl aliquots of culture media for 2 hours, 100 μl of Horseradish Peroxidase Conjugated Osteocalcin Antibody Solution for 1 hour, and 100 μl of Substrate Solution for 15 minutes; all incubation were performed at room temperature. The reaction was stopped with 100 µl of Stop Solution and absorbance was read at 450 nm using the plate reader SYNERGYMx (BioTek) equipped with Gen 5 1.09 software. The amount of released osteocalcin was calculated using the osteocalcin standard curve and expressed in “ng/ml”.

### Tissue formation

After 5 weeks in culture in both the SATE bioreactor and static conditions, samples were harvested to study tissue formation and penetration. Samples were washed in DPBS at room temperature for 5 min, fixed in 4% paraformaldehyde in DPBS (Santa Cruz Biotechnology, Dallas, TX, #SC-281692) at 4 °C for 2 days, decalcified in Immunocal (Decal Chemical Corp., Tallman, NY, #1440) for 4 days at 4 °C, then dehydrated through graded concentrations of ethanol prior to paraffin embedding. Sections (5 µm in thickness) were then cut at 3 different levels (25%, 50% and 75%), mounted on glass slides, and stained with hematoxylin and eosin (HE). Histological slides were then digitalized using the ScanScope^®^ GL scanner (Aperio, Vista, CA) at 20x magnification at standard resolution. Images were collected using the Aperio ImageScope v12.1.0.0529 software, then exported in JPG format using the Extract Region tool. Scanned images of histological stain were processed in Adobe Photoshop: image levels were adjusted from 0 to 255 to 50–200 to enhance the contrast for viewing.

Histomorphometrical analyses were performed using Adobe Photoshop (Adobe) to calculate the percentage of newly formed tissue. The total section area and the empty area (not filled with tissue) were selected using the Quick Select Tool and masked in white, and the total number of pixels was counted using the Measurement tool. The proportion of tissue formation, referred as “Tissue area”, was calculated using the following equation (2):

2 $$Tissue\,area=\frac{Total\,area-Empty\,area}{Total\,area}\ast 100 \% $$

### Immunohistochemical analysis

Deposition of bone matrix proteins was analyzed using immunohistochemistry (IHC) staining at the end of culture period (5 weeks). Samples were prepared as described in the **Tissue formation** section. Sections (5 µm in thickness) were mounted on charged slides, deparaffinized by heating at 60 °C for 30 min, followed by incubation in CitriSolv (twice for 5 min), rehydrated with a graded series of ethanol washes (twice 100%, 95%, 70%, 50%; each for 2 min), incubated in deionized water (three times for 2 min), and washed in DPBS for 5 min. The sections were then incubated in citrate buffer (pH 6) at 90 °C for 30 min for antigen retrieval, washed in deionized water for 5 min, and incubated with 3% H_2_O_2_ in methanol for 30 min to block the endogenous peroxidase activity. Following a wash in DPBS for 5 min, sections were incubated with 1% normal horse serum in DPBS (Vectastain ABC kit Elite, #PK-6200 Universal) to block the non-specific binding and stained overnight at 4 °C in a humidified chamber with primary antibodies (all purchased from Millipore and diluted 1:500 in DPBS) against osteopontin (rabbit polyclonal anti-osteopontin, #AB1870), bone sialoprotein (rabbit polyclonal anti-BSP II, #AB1854), and osteocalcin (rabbit polyclonal anti-osteocalcin, #AB10911). Specific antigen detection was performed using the biotinylated secondary antibody and biotin/avidin complex (Vectastain ABC kit Elite, #PK-6200 Universal) diluted in DPBS according manufacturer’s instructions and via incubation with 3,3′-diaminobenzidine peroxidase substrate for 5 min (Vector DAB kit, #SK-4100). Sections were then counterstained with hematoxylin (Richard-Allan Scientific, #7211), dehydrated with a graded series of ethanol washes (50%, 70%, 95%, twice 100%; each for 2 min), incubated with citrisolv (twice 5 min), dipped into xylene, and coverslipped (Fisherbrand, #12-545-M) using a Permount mounting media (Fisher Chemicals Scientific, #SP15-100). Negative controls were performed following the same procedure but omitting either the primary or secondary antibody incubation. Stained slides were then digitalized as described in the **Tissue formation** section. Scanned images of immunohistochemical stains were processed in Adobe Photoshop: image levels were adjusted from 0 to 255 to 50–200 to enhance the contrast for viewing.

### Animation

The SATE animation was made in Adobe Inventor (Adobe Systems) using objects drawn in AutoCAD (Autodesk). Adobe Premiere Pro CC (Adobe Systems) was used for final editing and to add the music track, voice-over and subtitles.

### Statistical analysis

Statistical analysis was conducted using Prism 6 version 6.0e. Student’s t-test was used for comparisons between groups. All results are shown as means and ±standard deviations. Differences were considered statistically significant when the p value was less than 0.05.

### Ethics statement

All methods were carried out in accordance with relevant guidelines and regulations.

All experimental protocols were approved by The New York Stem Cell Foundation Research Institute Committee.

Cells were derived from de-identified subjects and no informed consent was necessary for their use in this study.

## Electronic supplementary material

Supplementary Figures, Tables and Captions

Video S1

Video S2

Video S3

Video S4
