# Supplementary material for: Segmental Additive Tissue Engineering
Source: Sci Rep. 2018 Jul 18;8:10895. doi: 10.1038/s41598-018-29270-4 (PMC6052158; doi:10.1038/s41598-018-29270-4)
Supplement: Supplementary file 1 — Supplementary Figures, Tables and Captions [file 41598_2018_29270_MOESM1_ESM.pdf]

Segmental Additive Tissue Engineering

Martina Sladkova[1], Rawan Alawadhi[1], Rawan Jaragh Alhaddad[1], Asmaa Esmael[1], Shoug Alansari[1], Munerah Saad[1], Jenan Mulla Yousef[1], Lulwa Alqaoud[1], Giuseppe Maria de Peppo[1]

The New York Stem Cell Foundation Research Institute, New York, NY, USA[1]

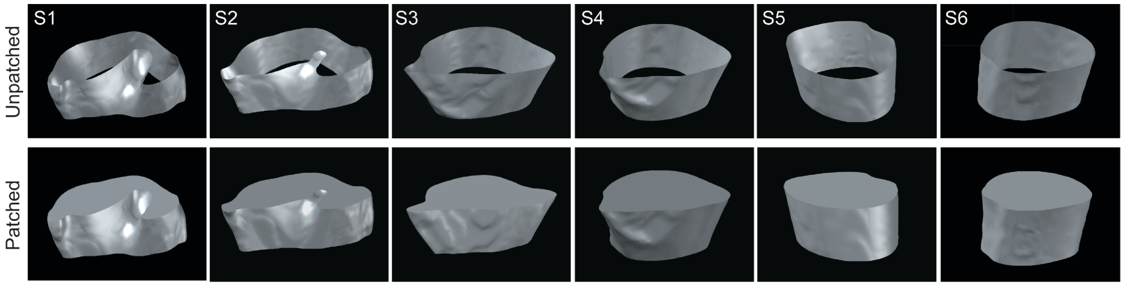

Figure S1. Segment patching. Patching of the virtual upper six segments (S1-S6) generated via partitioning of the digital model of the rabbit femur (images not in scale). Patched segments are used to assist the manufacturing of customized perfusion insert and biomaterial scaffolds.

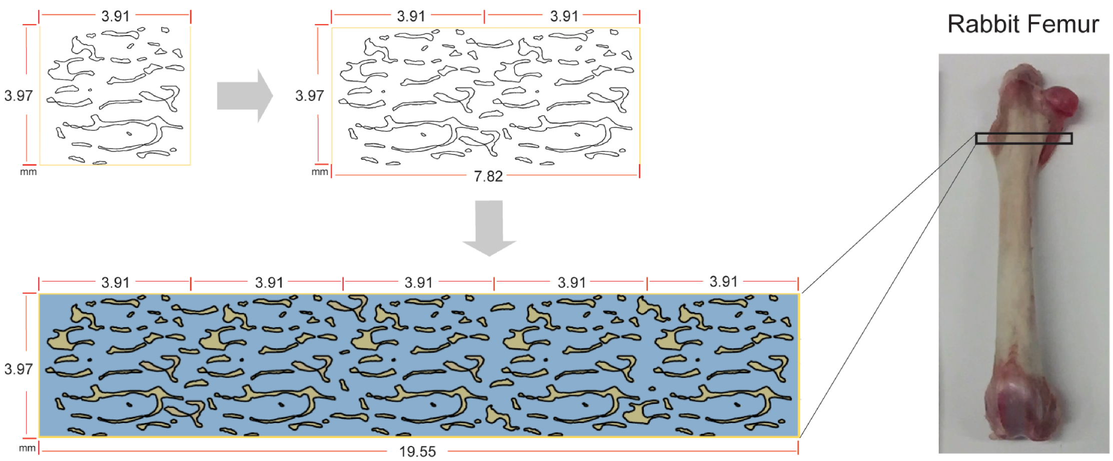

Figure S2. Production of a cross-section 2D image of decellularized bone scaffold for simulation studies. A 2D image representing the trabecular architecture of decellularized bone scaffolds was generated via microcomputed tomography, and used to reproduce a wider image with a size corresponding to the diameter of segment 1 (largest segment engineered in this study) generated from partitioning of the rabbit femoral model. Abbreviations: 2D, two-dimensional.

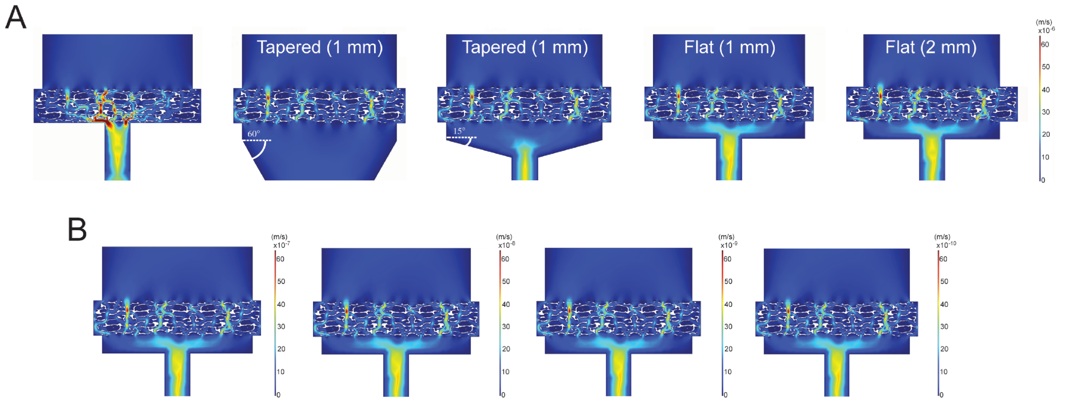

Figure S3. Flow dynamics simulation studies. (A) In silico simulation results showing the effect of an equilibration chamber, and its shape and size, on the perfusion dynamics throughout a 2D approximation of a discoidal bone graft (5 mm in height and 20 mm in diameter) at a given inlet velocity (10E-5 m/s). (B) In silico simulation results showing the effect of different inlet velocities (10E-6-10E-9 m/s) on the fluid dynamics throughout a 2D approximation of a discoidal bone graft using select equilibration chamber geometry (flat bottom and 1 mm reduction in diameter). Abbreviations: 2D, two-dimensional.

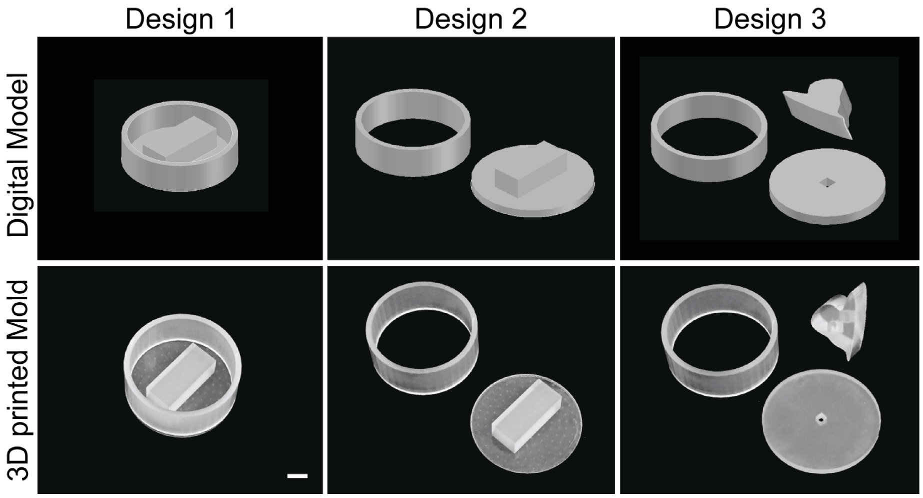

Figure S4. Mold design optimization for the casting of perfusion inserts. Different designs were tested for the production of customized perfusion inserts. Only design 3, which consists of 3 independent parts assembled together, enables the flawless production of perfusion inserts with complex geometry. Scale bar: 5 mm.

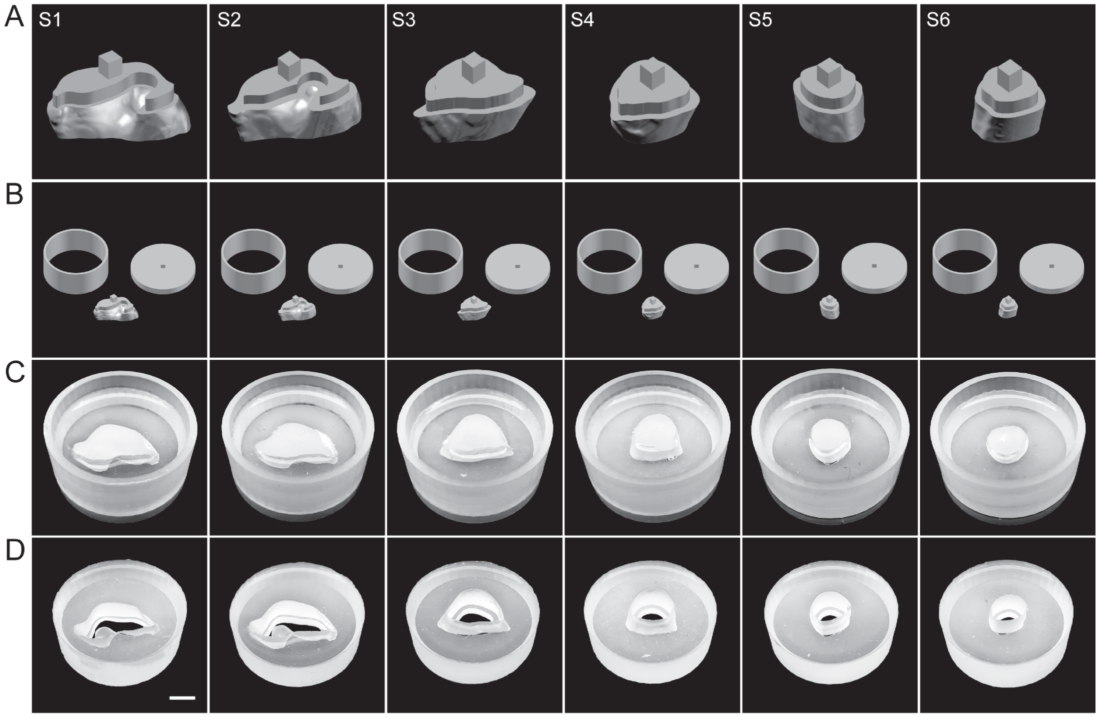

Figure S5. Casting of perfusion inserts. (A) Reshaped models of the six patched defect segments (S1-S6) including an offset for the production of the equilibration chambers and a plug for mold assembling. (B) Digital models of the molds for casting the customized perfusion inserts. (C) 3D printed molds assembled and filled with PDMS. (D) Customized perfusion inserts for direct perfusion culture in bioreactors. Scale bar: 5 mm. Abbreviations: PDMS, polydimethylsiloxane.

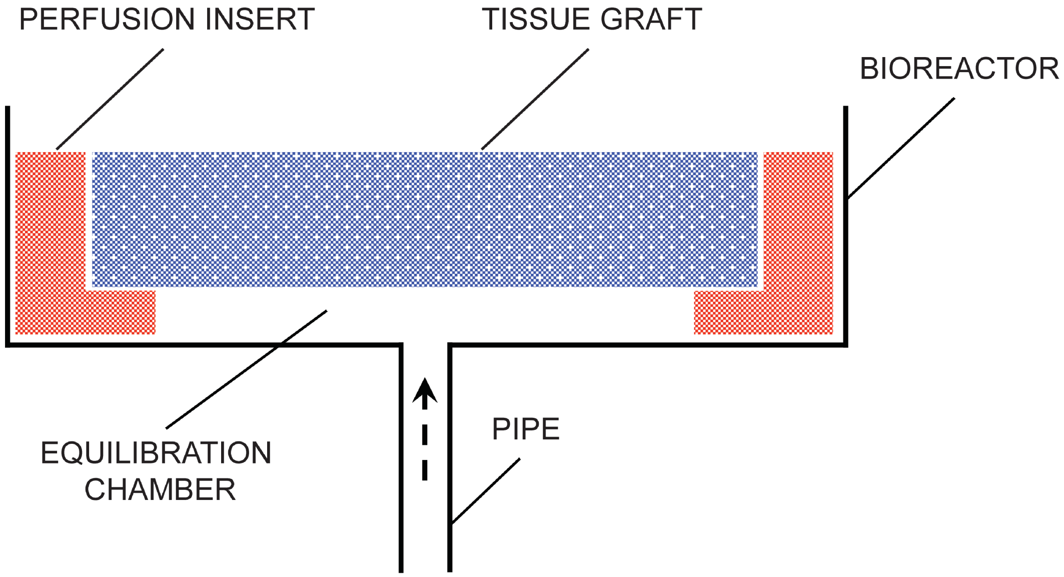

Figure S6. Equilibration chamber. Schematic of a cross-section of the perfusion system showing the equilibration chamber built within the perfusion inserts and beneath the tissue graft. The equilibration chamber allows redistribution of the medium exiting the pipe and guarantees uniform perfusion of the grafts irrespective of their size and geometry.

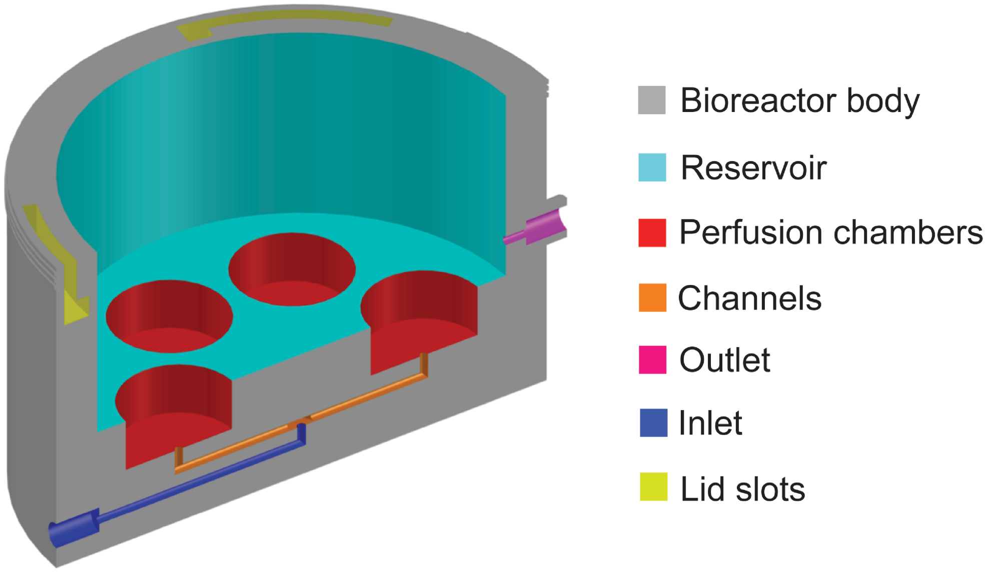

Figure S7. SATE Perfusion bioreactor. Realistic view of a cross section of the SATE perfusion bioreactor showing the configuration of the channel system and other elements.

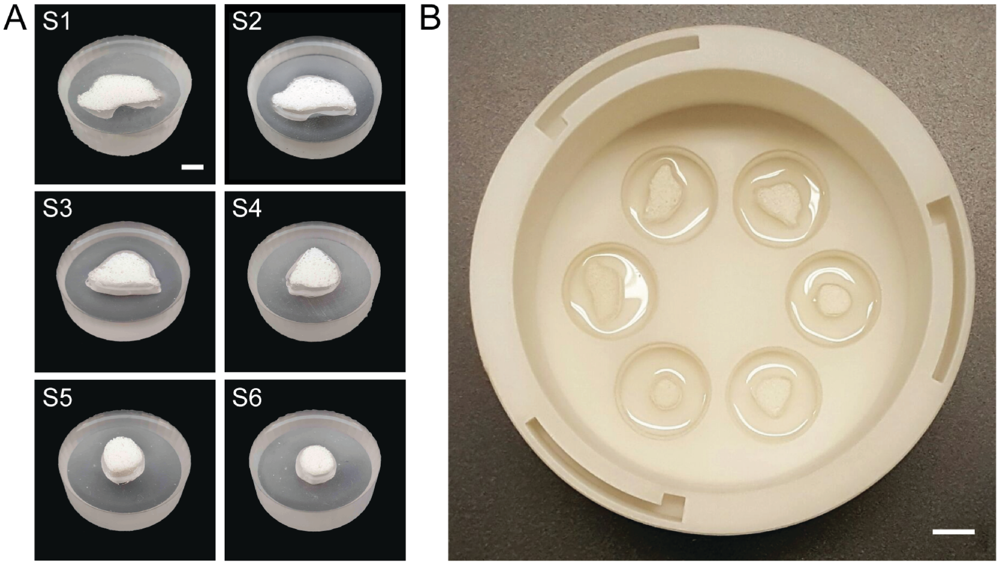

Figure S8. SATE perfusion bioreactor, perfusion inserts and scaffolds. (A) Customized decellularized bone scaffolds corresponding to the six defect segments (S1-S6) placed in their respective perfusion inserts. Scale bar: 5 mm. (B) Scaffold-perfusion insert constructs positioned in each chamber of the SATE perfusion bioreactor. Scale bar: 10 mm. Abbreviations: S, segment.

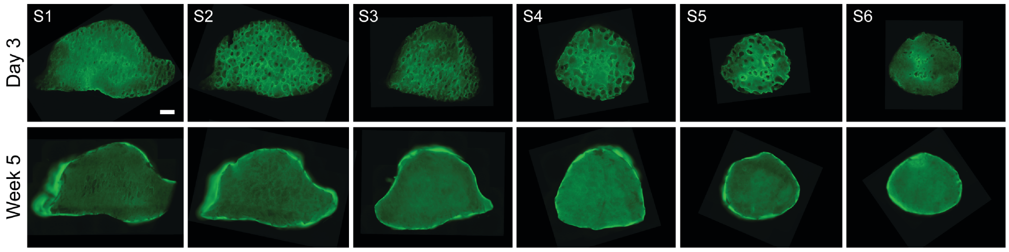

Figure S9. Additional data on cell seeding and growth. Epifluorescence mosaic micrographs showing distribution and viability of 1013A-derived mesenchymal progenitor cells stained with fluorescein diacetate (FDA; green) onto decellularized bone scaffolds corresponding to the six defect segments (S1-S6) 3 days after seeding, and 5 weeks after culture in static conditions. Scale bar: 2 mm. Abbreviations: S, segment.

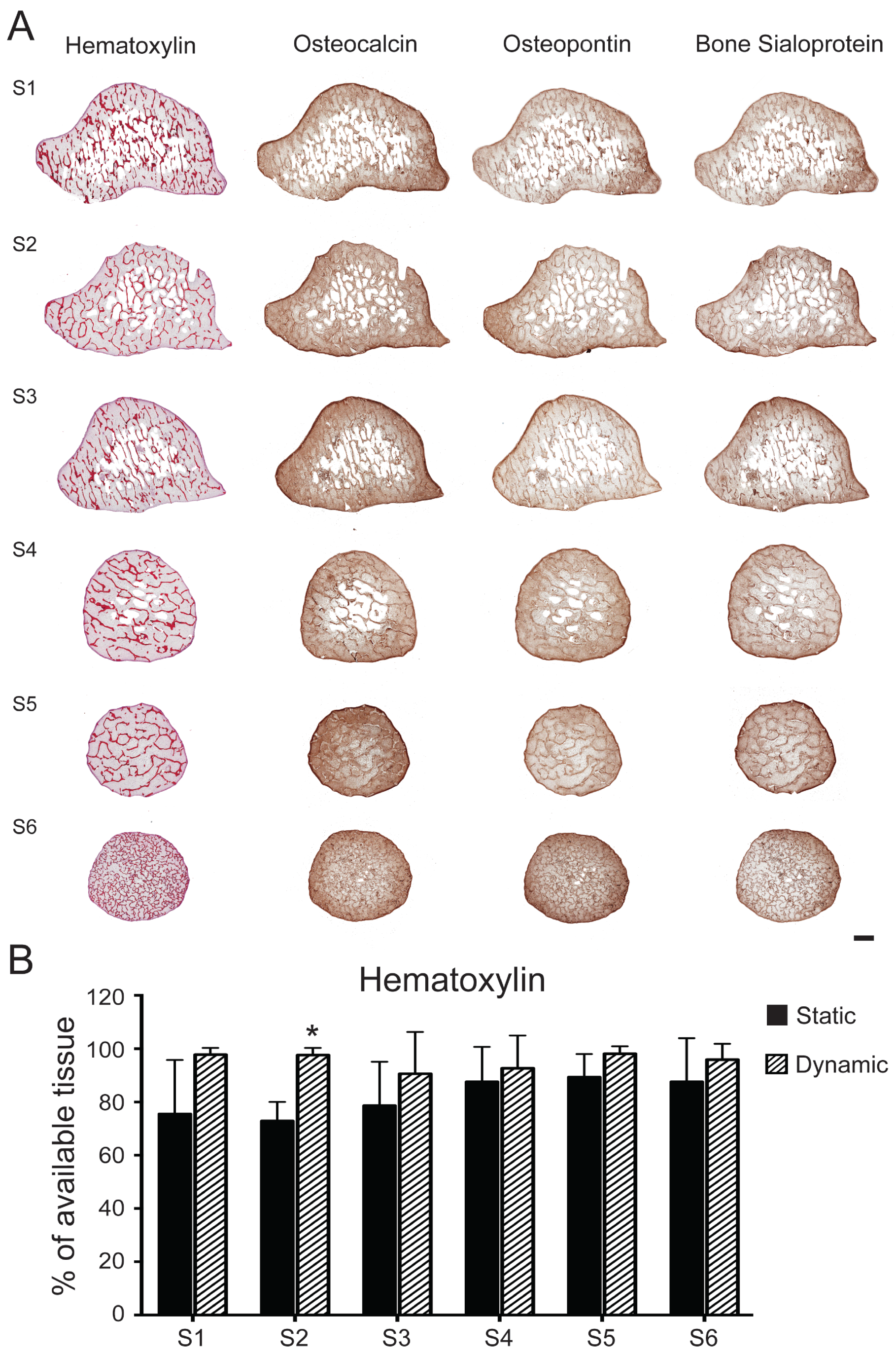

Figure S10. Additional data on tissue formation. (A) Histological analysis of samples corresponding to the six defect segments (S1-S6) cultured in static conditions. Samples are stained with hematoxylin/eosin, which stains cell nuclei blue and extracellular matrix pink. Scale bar: 2 mm. (B) Immunohistochemical analysis of samples corresponding to the six defect segments (S1-S6) cultured in static conditions. Scale bar: 2 mm. (C) Quantification of hematoxylin/eosin staining for samples corresponding to the six defect segments (S1-S6) cultured under static and dynamic conditions in bioreactors. Data represent averages ± SD (n = 3, Student's t-test, $P < 0.05$; * denotes significant difference to static conditions). Abbreviations: S, segment.

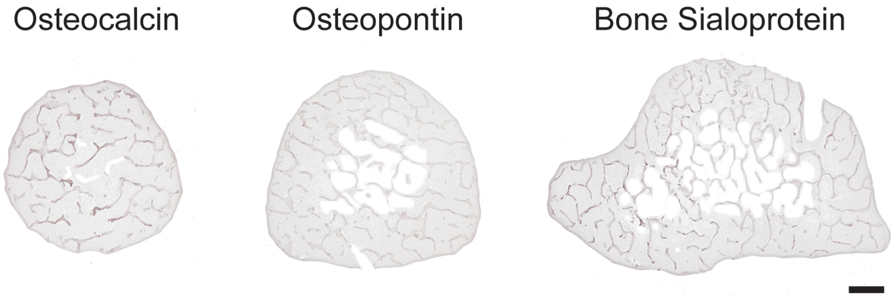

Figure S11. Additional data on immunohistochemical staining. Control sample staining negative for osteocalcin (left), osteopontin (middle) and bone sialoprotein (right) counterstained with hematoxylin. Samples correspond to segment 5 (left), segment 4 (middle), and segment 2 (right) from static cultures, respectively. Scale bar: 2 mm.

Table S1. Dimensions of the different SATE perfusion bioreactor elements and their volume.

	**Radius (mm)**	**Height (mm)**	**Length (mm)**	**Volume (mm^3)**
Reservoir	50	45	-	353429
Perfusion chamber	13	10	-	5309.29
Channels/Manifold	1	-	30	94.25
Manifold Hub	3	2	-	56.55
Outlet	1	-	5	15.71
Outlet fitting	3	-	10	282.74
Inlet	1	-	60	188.5
Inlet fitting	3	-	10	282.74
Lid	60	10	-	-

Table S2. Number of cells required per each scaffold.

	Total Volume (mm^3)	Void Volume (mm^3) *	Number of cells
Segment 1	410.57	328.46	8.17E+6
Segment 2	431.84	345.47	8.59E+6
Segment 3	393.60	314.88	7.83E+6
Segment 4	313.91	251.13	6.24E+6
Segment 5	221.89	177.51	4.41E+6
Segment 6	214.96	171.96	4.28E+6

* Void volume was calculated by estimating a scaffold porosity of 80%
